# IL-6, calcium, salivary amylase activity and cortisol as a salivary biomarker-combination associated with obesity: a pilot study

**DOI:** 10.3389/fdmed.2025.1725865

**Published:** 2026-01-09

**Authors:** H. Al Habobe, E. B. Haverkort, K. Nazmi, L. K. Van Nieukerken, V. E. A. Gerdes, F. J. Bikker, R. H. H. Pieters

**Affiliations:** 1 Research Group Innovative Testing in Life Sciences and Chemistry, Research Centre for Healthy and Sustainable Living, University of Applied Sciences Utrecht, Utrecht, Netherlands; 2 Institute for Risk Assessment Sciences, Faculty of Veterinary Medicine, Utrecht University, Utrecht, Netherlands; 3 Department of Oral Biochemistry, Academic Centre for Dentistry Amsterdam (ACTA), University of Amsterdam and VU University Amsterdam, Amsterdam, Netherlands; 4 Research Group Innovations in Preventive Care, University of Applied Sciences Utrecht, Utrecht, Netherlands; 5 Department of Bariatric Surgery, Spaarne Gasthuis Hospital, Hoofddorp, Netherlands; 6 Department of Vascular Medicine, Amsterdam University Medical Center (UMC), Amsterdam, Netherlands

**Keywords:** calcium, cortisol, IL-6, obesity, sAA, saliva

## Abstract

**Introduction:**

As obesity continues to escalate to pandemic levels worldwide, innovative approaches for early diagnosis, risk stratification, and disease monitoring are urgently needed. Saliva presents a promising non-invasive method for biomarker-based screening in obesity.

**Objective:**

This study aimed to utilize a multi-biomarker approach to explore associations between salivary biomarkers and obesity. This was done by measuring a pre-selected panel of obesity-related salivary biomarkers and comparing their levels between individuals with and without obesity.

**Methods:**

Unstimulated saliva was collected from 57 individuals, including 27 individuals diagnosed with obesity (BMI ≥ 30 kg/m^2^) and 30 non-obese controls (BMI < 30 kg/m^2^). Various biochemical techniques were used to quantify salivary total protein content, *α*-amylase activity (sAA), cortisol, interleukin 6 (IL-6), mucin 5B (MUC5B), albumin and calcium ions (Ca^2+^).

**Results:**

The results indicated lower Ca^2+^, sAA, and MUC5B levels (*P* < 0.05) and higher IL-6 and cortisol levels (*P* < 0.05) in the obese group compared to non-obese controls. In the obese group, Ca^2+^ correlated positively with most biomarkers, with sAA (r = 0.632, *P* < 0.05) and IL-6 (r = 0.449, *P* < 0.05) showing the strongest associations.

**Conclusions:**

In conclusion, this study highlights IL-6, Ca^2+^, sAA, and cortisol as a potential salivary biomarker-combination associated with obesity warranting further investigation. The observed changes in the salivary biomarker levels of the obese group may reflect underlying metabolic dysregulations, highlighting the advantage of a multi-biomarker approach to better capture early metabolic and inflammatory processes associated with obesity. To further validate these findings, large clinical studies with diverse, well-matched cohorts, as well as longitudinal studies, are needed.

## 1 Introduction

With its rising prevalence across diverse populations, obesity has emerged as a complex, multifactorial condition that affects a large segment of the global population. It is characterized by the accumulation of excess body fat, leading to chronic inflammation and significant health risks, including cardiovascular disease, cancer, type 2 diabetes mellitus, chronic obstructive pulmonary disease, dementia, and periodontitis (1–6). Despite the significant health risks associated with obesity, individuals often seek medical help only after complications become apparent. This is often due to the asymptomatic nature of the early metabolic and low-grade inflammatory changes that underlie obesity, which can easily go unnoticed without routine monitoring. As a consequence, preventive opportunities for early intervention are frequently missed, allowing the condition to progress to a more advanced harder-to-treat stage.

To address this challenge, the use of biomarkers as quantifiable biological indicators may be helpful to identify obesity-related metabolic dysregulations before symptoms become evident, enabling earlier prevention and intervention. Blood has traditionally been the primary and preferred medium for biomarker analysis in clinical research. However, increasing attention has turned towards saliva as non-invasive sampling method that does not require medical personnel (7–12). In line, numerous studies have utilized salivary biomarkers in exploring systemic conditions including obesity (13–21).

Yet, despite the growing number of studies on salivary biomarkers, research in the field of obesity primarily focuses on identifying novel biomarkers, examining single biomarkers, or studying biomarkers within a single category, such as proteins, lipids, or hormones (22–26). While these approaches may reveal significant differences between obese and non-obese individuals, they often fall short of providing a comprehensive understanding of the complex metabolic and inflammatory processes underlying obesity. This gap in salivary research is significant, as obesity is a multifactorial condition characterized by complex interactions between metabolic, inflammatory and enzymatic pathways (27–29). In contrast, a multi-biomarker approach that includes different biomarker categories is possibly more likely to retrieve a more comprehensive difference between obese and non-obese individuals. Moreover, we consider it plausible to hypothesize that this may lead to a better understanding of the underlying metabolic dysregulations in obesity. Furthermore, it holds promise for improving early detection, prevention, and targeted interventions.

Thus, this pilot study aimed to utilize a multi-biomarker approach to explore associations between salivary biomarkers and obesity. This was done by measuring a pre-selected panel of obesity-related salivary biomarkers in individuals with obesity and comparing their levels to those without obesity. The selection of candidate biomarkers was primarily based on including a variety of biomarkers from different categories, guided by findings from existing literature and our own work (30–32). This includes: the inflammatory marker IL-6, Ca^2+^ ions, total protein content, and cortisol, all of which have been reported to be elevated in individuals with obesity (33–37). Also, sAA was included, as it has been found to be reduced in individuals with obesity (20). In addition, MUC5B was included, as it serves as an important nutrient for oral bacteria that may modulate inflammation and potentially impact the presence or absence of inflammatory markers in the oral cavity (38). Albumin was also included as a biomarker to determine whether inflammation observed in obese individuals could be attributed to oral tissue damage. Elevated salivary albumin levels were previously associated with inflammatory conditions such as periodontitis (39), which, in turn, has been found to be associated with obesity (40).

As far as we are aware, no previous cross-sectional study has assessed the salivary profile of individuals with obesity using a multi-biomarker approach encompassing a diverse set of biomarkers across different functional categories.

## 2 Materials and methods

### 2.1 Study population

All procedures were performed in compliance with relevant laws and institutional guidelines and have been approved by the appropriate institutional committee(s). This study was approved by the Utrecht University of Applied Sciences Ethical Board for Human Studies (no. 171-000-2022), as well as the Ethical Board of Spaarne Gasthuis Hospital, Hoofddorp, the Netherlands, and was conducted in accordance with the principles of the Declaration of Helsinki and STROBE guidelines for human observational cross-sectional studies. The privacy rights of human subjects have been observed and written informed consent was obtained from all participants. Participants without obesity were recruited during their visit to the dental department of the University Teaching Clinic, while participants with obesity were recruited during their visit to the bariatric department of Spaarne Gasthuis Hospital. Participants were excluded if they had oral conditions such as gingivitis or periodontitis, were pregnant, smoked, or used medications with anticholinergic properties, as these factors are known to influence salivary composition (41–45). A total of 57 participants were included, of whom 27 had a clinical diagnosis of obesity [20 females and 7 males, age 43 ± 14 years, body mass index (BMI) > 35 kg/m^2^] and 30 were non-obese controls (17 females and 13 males, age 31 ± 13 years, BMI < 30 kg/m^2^).

### 2.2 Saliva collection and storage

To standardize the collection of saliva samples and minimize variability, participants were instructed to refrain from tooth brushing, food and fluid intake (except for water), physical activity, and the use of lip cosmetics for at least one hour prior to sampling. Unstimulated saliva was collected following a standardized protocol (30, 46). Briefly, samples were obtained between 9:00 and 12:00 a.m., with participants seated comfortably in an upright posture, their heads slightly inclined forward to allow pooling of saliva. At 30-second intervals, saliva was expectorated into pre-weighed, sterile 50 mL polypropylene tubes labeled in advance. Directly after sampling, the tubes were reweighed to determine salivary flow rate. Throughout the collection process, samples were kept on ice. Saliva was then centrifuged at 2,075 × g for 5 min at 4 °C to remove any cellular components. The supernatant was subsequently aliquoted into 1.5 mL Eppendorf tubes and stored at −80 °C pending analysis.

### 2.3 Measurement of total protein concentration in saliva

Total protein concentration in saliva was determined, essentially as previously described (30), using the Bicinchoninic Acid (BCA) Protein Assay Kit (Thermo Fisher Scientific, Bleiswijk, the Netherlands). Saliva samples were pre-diluted at a 1:3 ratio in phosphate-buffered saline (PBS). A standard curve was prepared using serial dilutions of bovine serum albumin, ranging from 0 to 1,500 µg/mL. For each sample and standard, 20 µL was mixed with 180 µL of protein detection reagent in 96-well microplates (Greiner Bio-One, Frickenhausen, Germany). Absorbance was measured at 562 nm using a Multiskan FC plate reader (Thermo Scientific, Waltham, MA, USA), and total protein levels were expressed in mg/mL

### 2.4 Measurement of sAA

sAA activity was assessed via a colorimetric enzymatic method, essentially as previously described (30). Samples were diluted 1:100 in Millipore water, and 10 µL of each sample was combined with 90 µL of 2-chloro-4-nitrophenyl-α-D-maltotrioside (Sigma-Aldrich, Zwijndrecht, the Netherlands), a substrate specific for *α*-amylase. The production rate of the breakdown product, 2-chloro-4-nitrophenol, was recorded at 405 nm over a 15 min period using a Multiskan FC spectrophotometer. Enzyme activity was expressed in U/mL, based on calibration against a known *α*-amylase standard (1 U).

### 2.5 Measurement of salivary cortisol concentration

Cortisol levels in saliva were quantified using a competitive ELISA kit (Thermo Fisher Scientific), in accordance with the manufacturer's instructions. Undiluted samples were used. A standard curve ranging from 0 to 3,200 pg/mL was prepared. Absorbance was read at 450 nm using a Multiskan FC plate reader. Cortisol concentrations were calculated using four-parameter logistic regression and reported in pg/mL.

### 2.6 Measurement of salivary IL-6 concentration

IL-6 concentrations were determined using a high-sensitivity ELISA kit (Abcam B.V., Amsterdam, the Netherlands), following the protocol provided by the manufacturer. Undiluted samples were used. A standard curve ranging from 0 to 50 pg/mL was prepared. Absorbance was read at 450 nm using a Multiskan FC reader, and concentrations were computed using four-parameter logistic regression, reported in pg/mL.

### 2.7 Measurement of salivary MUC5B concentration

Salivary MUC5B levels were determined by ELISA, essentially as previously described (30). Saliva samples were diluted 1:800 in coating buffer (0.1 M NaHCO₃, pH 9.6). A standard curve of MUC5B (0–6 µg/mL) was prepared. Samples and standards were coated to NUNC MaxiSorp™ 96-well high-binding ELISA plates (Thermo Fisher Scientific) and incubated overnight at 4 °C. MUC5B was detected using the F2 monoclonal antibody specific to MUC5B. Absorbance was recorded at 492 nm using the Multiskan FC plate reader, and MUC5B concentrations were determined using four-parameter logistic regression in mg/mL.

### 2.8 Measurement of salivary albumin concentration

Albumin concentrations were assessed using a sandwich ELISA, essentially as previously described (30). ELISA plates (Thermo Fisher Scientific) were coated overnight at 4 °C with 100 µL of a 1:8,000 diluted rabbit anti-human albumin antibody (DAKO, cat# A0001) in PBS with 0.1% Tween-20. Saliva samples, diluted 1:1,000 in the same buffer, were added in 100 µL volumes. An albumin standard was added on each plate. Absorbance was recorded at 492 nm using the Multiskan FC plate reader. Albumin concentrations were determined via four-parameter logistic regression and expressed in mg/mL.

### 2.9 Measurement of salivary calcium Ion concentration

Salivary calcium ion (Ca^2+^) levels were quantified using a capillary electrophoresis system (CAPEL-205, Lumex Instruments Canada, Mission, BC, Canada), essentially as previously described (30). Samples were diluted 1:10 in Millipore water and analyzed using a cation standard solution containing Ca^2+^. Separation was achieved using a background electrolyte buffer (BGE) composed of 20 mM benzimidazole, 5 mM tartaric acid, and 2 mM 18-Crown-6 (kit no. 0300001550), as specified by the manufacturer. Quantification was performed using external calibration, and data analysis was conducted with ELFORUN-205 software (Envico, Zoeterwoude, the Netherlands). Calcium concentrations were expressed in millimolar (mM).

### 2.10 Statistical analysis

#### 2.10.1 Sample size calculation

The required sample size for this pilot study was determined *a priori* using a power analysis conducted in G*Power version 3.1.9 (Faul et al., 2009) (47), based on the primary aim of comparing salivary biomarker concentrations between obese and non-obese individuals. Assuming a large effect size (Cohen's d = 0.8), a significance level of *α* = 0.05, and a statistical power of 0.80, the minimum required sample size was estimated at 26 participants per group (total *n* = 52). To account for potential dropout or missing data (∼10%), the target sample size was increased to 29 participants per group (*n* = 58). Ultimately, the study included 30 non-obese controls and 27 obese participants (total *n* = 57), which was expected to be sufficient to maintain adequate statistical power for this pilot and detecting meaningful group differences.

#### 2.10.2 Data analysis

All statistical analyses were performed using IBM SPSS Statistics version 28.0 (IBM Corp., Armonk, NY, USA). Non-parametric tests were applied. Descriptive statistics are presented as medians with interquartile ranges (IQR). Group comparisons between obese and non-obese participants were conducted using the Mann–Whitney *U*-test. To assess associations between salivary biomarkers in unstimulated saliva of individuals with obesity, Spearman's rank correlation coefficient was used. This non-parametric measure evaluates the strength and direction of monotonic relationships, with values ranging from −1 (perfect negative) to +1 (perfect positive); a coefficient of 0 indicates no monotonic association. A two-tailed *p*-value < 0.05 was considered statistically significant. Uncorrected *p*-values were reported, and the correlations are interpreted as indicative rather than confirmatory findings. The effect size was calculated using the Mann–Whitney *U*-test formula, $r=\frac{|z|}{\sqrt{n}}$, where *z* is the standardized test statistic from the Mann–Whitney *U*-test and *n* is the total sample size. According to Cohen's guidelines, *r* values of approximately 0.10, between 0.30-0.50, and above 0.50 represent small, medium, and large effects, respectively.

#### 2.10.3 Missing datapoints

Missing data were observed for three biomarkers: MUC5B (11 cases), albumin (13 cases) and cortisol (21 cases), due to insufficient remaining sample volume. These missing values were classified as missing completely at random (MCAR) (48), as they resulted from insufficient sample volume. Therefore, a complete case analysis was performed for each biomarker, and no imputation methods were applied. MCAR data has the statistical advantage that analyses remain unbiased, meaning that correlation coefficients, *p*-values and other parameter estimates are not distorted by the absence of data (48).

## 3 Results

### 3.1 Characteristics of the study population

Table 1 presents the demographic and anthropometric characteristics of the study population, divided into obese and non-obese groups (30). The obese group comprised of 27 participants (20 females, 7 males) with a mean age of 43 ± 14 years and a mean BMI of 42 ± 5 kg/m^2^, while the non-obese group included 30 participants (17 females, 13 males) with a mean age of 31 ± 13 years and a mean BMI of 24 ± 4 kg/m^2^. The table highlights the significant differences in BMI and age between the two groups, with the obese group being older and exhibiting higher BMI values than the non-obese group.

**Table 1:** Demographic characteristics of the study population.

Characteristics	Obese	Non-obese	*p*-value^a^
Number of participants	27	30	>0.05
Age in years *(Mean* *±* *SD)*	43 ± 14	31 ± 13	<0.05
Sex (F, M)	F = 20; M = 7	F = 17; M = 13	
BMI (kg/m^2^) *(Mean* *±* *SD)*	42 ± 5	24 ± 4	<0.05

BMI, body mass index; F, female; M, male

a Mann–Whitney *U*-Test; *P* < 0.05 is considered significant

### 3.2 Comparison of unstimulated salivary flow rate

Table 2 shows the unstimulated salivary flow rate (mL/min) for obese and non-obese individuals (30). The median salivary flow rate in obese individuals was 0.37 mL/min (IQR: 0.25–0.50), compared to 0.28 mL/min (IQR: 0.18–0.63) in non-obese individuals. The Mann–Whitney *U*-test indicated no statistically significant difference between the groups (*p* > 0.05).

**Table 2:** Unstimulated salivary flow rate (mL/min) in individuals with .

Obesity and without obesity		
Group	Median (IQR)	*p*-value^a^
Obese individuals	0.37 (0.25–0.50)	(a) > 0.05
Non-obese individuals	0.28 (0.18–0.64)	

IQR, interquartile range

a Mann–Whitney *U*-Test; *P* < 0.05 is considered significant.

### 3.3 Comparison of salivary biomarker levels

Table 3 presents the comparison of salivary biomarkers between individuals with and without obesity. Several significant differences were observed, most of which were accompanied by medium to large effect sizes. Individuals with obesity showed significant lower Ca^2+^ levels, with a large effect (r = 0.8), as well as lower sAA (r = 0.3) and MUC5B levels (r = 0.7), both reflecting medium to large effects. In contrast, IL-6 and cortisol concentrations were significantly higher in individuals with obesity, each showing medium-to-large effect sizes (r = 0.6 and r = 0.5, respectively). No significant differences were observed for total protein content or albumin, and effect sizes were small (r = 0.1).

**Table 3:** Comparison of measured salivary biomarker levels between individuals with and without obesity.

Biomarker category	Biomarker	Obese	Non-Obese	*p*-value^a^	Effect
					size
		Median (IQR)	Median (IQR)		*r*
Ion	Ca^2+^ (mM)	0.061 (0.037–0.11)	0.28 (0.23–0.34)	<0.05	0.8
Enzyme activity	sAA (U/mL)	17 (4.9–45)	33 (15–71)	<0.05	0.3
Proteins	Total protein (mg/mL)	1.3 (0.9 -2.3)	1.3 (0.9–1.5)	0.512	0.1
	MUC5B (mg/mL)^b^	3.4 (1.9–6.4)	18 (10–23)	<0.05	0.7
	Albumin (mg/mL)^b^	0.044 (0.023–0.072)	0.041 (0.023–0.050)	0.283	0.1
Cytokine	IL-6 (pg/mL)	5.4 (3.1–7.8)	1.2 (0.5–1.7)	<0.05	0.6
Hormone	Cortisol (pg/mL)^b^	290 (174–372)	111 (47–206)	<0.05	0.5

a Mann–Whitney *U*-Test; *P* < 0.05 is considered significant; IQR, interquartile range.

Effect size r for the Mann Whitney *U*-Test; *r* < 0.3 small effect; *r* between 0.3–0.5 medium effect; *r* > 0.5 large effect.

b Biomarkers with missing data: MUC5B (11 cases), Albumin (13 cases) and cortisol (21 cases).

### 3.4 Intercorrelations among salivary biomarkers in obesity

Table 4 summarizes the significant inter-biomarker correlations observed in the obese and control groups. In the obese group, several biomarkers demonstrated moderate to strong positive associations. Calcium showed significant correlations with sAA, total protein, albumin, and IL-6 (*ρ* ranging from 0.448 to 0.657). In addition, sAA correlated positively with total protein and albumin, and albumin was significantly associated with total protein. No corresponding significant correlations were observed for these biomarker pairs in the control group.

**Table 4:** Significant inter-biomarker correlations in obese and control groups (spearman's *ρ*).

Biomarker pair	Obese (*ρ*)	Control (*ρ*)
Ca^2+^ vs. sAA	0.657*	
Ca^2+^ vs. Total protein	0.471*	
Ca^2+^ vs. MUC5B		0.413*
Ca^2+^ vs. Albumin	0.605*	
Ca^2+^ vs. IL-6	0.448*	
sAA vs. Total protein	0.797*	
sAA vs. MUC5B		0.587*
sAA vs. Albumin	0.643*	
Albumin vs. Total protein	0.547*	

* *p* < 0.05 is considered significant. Correlations that were non-significant in both the obese and control groups were omitted for clarity. The flow rate was significantly correlated with Ca^2+^.

In contrast, only two biomarker relationships reached significance in the control group: Ca^2+^ correlated positively with MUC5B (*ρ* = 0.413), and sAA correlated with MUC5B (*ρ* = 0.587). These associations were not present in the obese group. Finally, salivary flow rate was significantly correlated with Ca^2+^, while no other significant associations with flow rate were detected.

## 4 Discussion

This pilot study applied a multi-biomarker approach to compare salivary profiles of individuals with and without obesity (30). Individuals with obesity exhibited higher salivary IL-6 and cortisol levels and lower Ca^2+^, sAA, and MUC5B levels, while salivary flow rate did not differ significantly between groups. Together, these findings suggest that obesity is associated with measurable alterations in salivary biochemistry that may reflect the inflammatory and metabolic state characteristic of excess adiposity.

The elevated IL-6 and cortisol levels observed in the obese group are consistent with the well-established pro-inflammatory and endocrine disturbances associated with adipose tissue dysfunction (33–37, 49–51). Reduced sAA activity aligns with earlier reports showing inverse relationships between amylase activity and measures of adiposity (20). Similarly, reduced Ca^2+^ levels parallel findings of altered mineral metabolism in obesity (34), although the biological significance of this association in saliva remains unclear. While several mechanisms may help contextualize these findings, interpretations remain hypothetical and lie beyond the scope of this pilot study. For example, circulating IL-6 may enter saliva through established transcellular or paracellular routes (52), and lower sAA or Ca^2+^ levels could relate to alterations in sympathetic activity, salivary gland function, or broader metabolic dysregulation. Interpretation of the MUC5B findings should be approached with caution. Although individuals with obesity showed lower MUC5B levels with large effect size, this study cannot determine whether this reflects changes in glandular secretion, mucin glycosylation, or microbiome-mediated degradation (38, 53–55). Because the F2 monoclonal antibody targets a single MUC5B-derived epitope, differences in antigen detection may not directly correspond to overall mucin abundance.

Taken together, our dataset highlights physiological alterations associated with obesity and provides a basis for future studies aimed at uncovering the underlying mechanisms. A strength of this study is the simultaneous assessment of inflammatory, hormonal, ionic, enzymatic, and mucin markers, enabling a more integrated overview than single-biomarker approaches (22–26). The combined alterations in IL-6, Ca^2+^, sAA, MUC5B and cortisol indicate a potential salivary biomarker pattern associated with obesity, underscoring the value of a multi-biomarker strategy for metabolic risk profiling.

### 4.1 Limitations

This study has several limitations that should be considered. First, the cross-sectional and exploratory design prevents conclusions about causality or temporal dynamics and kinetics of effects, particularly for biomarkers such as cortisol, underscoring the need for longitudinal studies to validate these associations. Second, differences between participants with regard to age and gender may also have influenced the results. Because age influences several salivary biomarkers, particularly cortisol and IL-6, the observed group differences may be partially attributable to age rather than obesity alone highlighting the importance of larger studies with more diverse and closely matched cohorts. In addition, as recruitment took place at a single obesity center and a university clinic, the generalizability of the findings may be limited. Finally, uncorrected *p*-values were reported due to the exploratory nature and limited sample size of this pilot study. As a result, the observed correlations should be interpreted with caution, as the absence of multiple-comparison adjustments may have increased the risk of Type I error. Although the missing values for cortisol were classified as missing completely at random, this may still have limited statistical power.

## 5 Conclusion

In conclusion, this study highlights IL-6, Ca^2+^, sAA, MUC5B and cortisol as a potential salivary biomarker-combination associated with obesity. The observed changes in biomarker levels may reflect underlying metabolic dysregulations, reinforcing the need for a multi-biomarker approach to better capture early metabolic and inflammatory processes associated with obesity. To further validate these findings, larger clinical studies with diverse and well-matched cohorts, as well as longitudinal studies, are needed. Additionally, comparison with blood measures as a reference standard will be crucial to further validate the clinical applicability of salivary biomarkers.

## Data Availability

The original contributions presented in the study are included in the article/Supplementary Material, further inquiries can be directed to the corresponding author.
